# Integrated analysis based on vesicle trafficking‐related genes identifying CNIH4 as a novel therapeutic target for glioma

**DOI:** 10.1002/cam4.5947

**Published:** 2023-04-16

**Authors:** Zhen Fang, Fangen Kong, Jia Zeng, Zichen Zhang, Yunzhi Wang, Yiping Wang, Jiajia Duan, Lei Chen, Jikai Wang, Fei Liu

**Affiliations:** ^1^ Department of Neurosurgery The Fifth Affiliated Hospital of Sun Yat‐sen University Zhuhai Guangdong China; ^2^ Guangdong Provincial Key Laboratory of Biomedical Imaging, The Fifth Affiliated Hospital Sun Yat‐sen University Zhuhai Guangdong China; ^3^ Guangdong Provincial Engineering Research Center of Molecular Imaging, The Fifth Affiliated Hospital Sun Yat‐sen University Zhuhai Guangdong China

**Keywords:** CNIH4, glioma, glioma stem cell, immunotherapy, prognosis, tumor immune microenvironment, vesicle trafficking

## Abstract

**Background:**

Vesicle trafficking is a highly important process in numerous human diseases, especially in the central nervous system dysfunctions. However, as a key component of vesicle trafficking‐related genes (VRGs), Cornichon family AMPA receptor auxiliary protein 4 (CNIH4) has not been systematically elucidated in glioma so far.

**Methods:**

Differentially expressed VRGs were selected using molecular signatures database (MSigDB), The Cancer Genome Atlas (TCGA), and Genotype‐Tissue Expression (GTEx) mRNA expression profiles. Further exploration of CNIH4 was determined using LASSO‐Cox regression algorithms. Then Kaplan–Meier (K‐M) plotter, receiver operating characteristic (ROC) curves, and multivariate Cox regression analyses were utilized to assess the independent significance of CNIH4 in the CGGA validation cohort. Functional exploration was performed with Gene Set Enrichment Analysis (GSEA) and then verified using a series of functional experiments in glioma cells. Finally, the consensus clustering algorithm was applied to identify clusters in glioma samples. After that, differences in prognosis, the tumor immune microenvironment (TIME), and therapy response were evaluated between clusters.

**Results:**

CNIH4 was shown to be overexpressed in malignant glioma variants and was frequently observed in GCSs and TMZ‐resistant cell lines. Higher CNIH4 levels were significantly related to poor outcomes and positively correlated with adverse clinicopathological characteristics. Survival analyses revealed CNIH4 as an independent risk factor that outperformed traditional measures. Enrichment analysis indicated that overactive CNIH4 significantly gathered in stem cell processes. Furthermore, functional assays of silencing CNIH4 expression suppressed stem cell‐like properties in vitro and inhibited tumorigenicity in vivo. Finally, the CNIH4‐enriched subgroup negatively modulated immunotherapeutic response and reflected lower chemotherapy sensitivity for glioma patients.

**Conclusion:**

Our study identified CNIH4 as a potential VRG that regulates tumor stemness, microenvironment immunity, and chemotherapy sensitivity. It may serve as a novel prognostic factor and a promising target against glioma therapy.

## 1 INTRODUCTION

Glioma is the most common and lethal CNS tumor, distinguished by its aggressive infiltrative form, therapy resistance, and rapid recurrence rate.^1^ Despite significant progress in molecular pathologic detection and comprehensive therapeutic strategies, the long‐term survival of advanced glioma remains unsatisfactory, with a 5‐year survival <10%.^2^ Numerous tumor‐specific genetic alterations such as typical isocitrate dehydrogenase (IDH) mutation and chromosome 1p/19q codeletion were routinely applied in diagnosis according to the 2021 World Health Organization (WHO) classification system, but whether these gene aberrations affect glioma progression remains debatable.^3^, ^4^ Therefore, the screen of pivotal biomarkers to be served as a diagnostic and therapeutic target in glioma is of great significance.

Increasing studies have demonstrated that vesicle trafficking intimately plays roles in many aspects of tumorigenesis, including tumor growth, invasion, migration, cell cycle regulation, angiogenesis, and even immune‐mediated functions.^5^ Cornichon family AMPA receptor auxiliary protein 4 (CNIH4), known as a central protein of vesicle trafficking family members, regulates vesicles transporting from their synthesis site to the functional site.^6^ Researches indicated that CNIH4 potentially increases colon cancer cell metastatic activity by forming a positive feedback loop with TMED9, GLI1, and TGFα.^7^ Besides, higher CNIH4 activity predicted poor prognosis in several human malignancies in recent investigations.^7^, ^8^, ^9^ However, the therapeutic application and molecular processes of CNIH4 in glioma have not been fully discussed so far.

In the present investigation, we determined the critical membership of CNIH4 in a prognostic model based on 8 VRGs. Then we identified the expression profile of CNIH4 in glioma tissues and cell lines using publicly available data. Increased CNIH4 expression was positively correlated with tumor progression, chemotherapy sensitivity, and microenvironment immunity. Functional annotation demonstrated typical accumulation of CNIH4 during EMT formation, stem cell differentiation, apoptosis, and tumor stroma processes. Furthermore, CNIH4 silencing was revealed to dramatically enhanced TMZ sensitivity and apoptosis, while inhibiting proliferation, migration /invasion, sphere‐formation, as well as tumorigenicity in glioma cells. Notably, CNIH4 outperformed established indicators in terms of predictive value. All these findings suggest that CNIH4 to be served as a potential candidate for glioma therapy.

## 2 MATERIALS AND METHODS

### 2.1 Data collection and processing

The Chinese Glioma Genome Atlas (CGGA, https://www.cgga.org.cn/), Gene Expression Omnibus (GEO, https://www.ncbi.nlm.nih.gov/geo/), and The Cancer Genome Atlas (TCGA, https://portal.gdc.cancer.gov/) were used to obtain transcriptome profile data with associated clinical annotations. Downloads from The Genotype‐Tissue Express (GTEx, https://gtexportal.org/) were used to replenish deficient normal tissue samples. A list of vesicle trafficking‐related genes was extracted from the MSigDB website. Data from RNA sequencing were log2‐transformed before being further processed. The “SVA” package was applied in advance to integrate the sequencing profile and reduce heterogeneity between batches from the CGGA (mRNAseq_693 & mRNAseq_325) and TCGA (TCGA‐GBM & TCGA‐LGG) sequencing databases, respectively.^10^ A total of 749 subjects from the CGGA and 666 from TCGA were finally included.

### 2.2 Construction of vesicle trafficking‐related prognostic signature

The “DESeq2” R package was applied to select differentially expressed genes (DEGs) from 666 TCGA samples and 5 adjacent normal samples. Adj *p*‐value <0.05 and |log2 fold change (FC)| > 2 were set as cut‐off values. Volcano mapping of differentially expressed VRGs was performed using the “Enhancedvolcano” R package. LASSO‐Cox regression analysis was utilized to eliminate gene collinearity and select prognostic candidates. Finally, an eight‐gene optimal prognostic model was constructed and the risk score for each patient was calculated as follows: risk score = βmRNA1 × mRNA1 expression + βmRNA2 × mRNA2 expression + · ···· + βmRNAn × mRNAn expression.

### 2.3 The Human Protein Atlas

Human Protein Atlas (HPA, https://www.proteinatlas.org/) is an available program designed to promote systematic exploration of the human proteome using antibody‐based proteomics.^11^ Our study compared the protein level of CNIH4 in glioma tissues and normal brain tissues by obtaining Immunohistochemistry (IHC) staining images from the HPA database. As previously stated, the protein expression level was determined by counting both the fraction of stained cells and the intensity of the staining.^12^

### 2.4 Gene Set Enrichment Analysis

Gene Set Enrichment Analysis (GSEA) is a computational tool that determines whether a set of genes exhibits significant and consistent variations across parameters.^13^ We employed GSEA to estimate biological hallmarks connected with CNIH4 expression and Gene Ontology (GO) analysis to annotate functional pathway activation. As previously stated, |NES| > 1, normalized *p*‐value <0.05, and FDR q‐value <0.25 denoted statistical significance.

### 2.5 Cell culture and transfection

Human astrocytes (HA) and glioma (LN229, T98, U251, and U87) cell lines were supplied by the Institute of Biochemistry and Cell Biology, Chinese Academy of Sciences. Cells were cultured in DMEM supplemented with 10% fetal bovine serum and 1% penicillin–streptomycin. All cells were cultured at 37°C with 5% CO2 in a humidified incubator. Lentiviral plasmids (pLV3‐CMV‐GFP‐Luc‐shCNIH4 and pLV3‐CMV‐GFP‐Luc‐shCtrl) from GenePharma Co, Ltd were co‐transfected into 293 T cells with three packaging plasmids. Following 48 h of viral fluid incubation, a culture medium containing 3.0 μg/mL puromycin (Solarbio) was replenished for at least 1 week before transduced cell collection.

### 2.6 Real‐time quantitative PCR (RT‐PCR) and western blotting

Total RNA was obtained from cells following the manufacturer's procedure using the Total RNA Kit I (Omega Bio‐Tek). The reverse transcription reaction of RNA was carried out with the aid of the HiScipt III reverse transcription kit (Vazyme). The real‐time quantitative polymerase chain reaction (RT‐PCR) was then applied using the ChamQ Universal SYBR qPCR kit (Vazyme). MRNA expression was normalized to β‐actin mRNA expression and quantified using the 2 −ΔΔCt technique. The following primers were used to detect CNIH4 and β‐actin specifically: CNIH4 forward: 5′‐TCAACTTACCTgTTgCCACTTg‐3′; CNIH4 reverse: 5′‐TCTgTTggATCAAACACTCCCA‐3′; β‐actin forward: 5′‐ggATTCCTATgTgggCgACgA‐3′; β‐actin reverse: 5′‐gCgTACAgggATAgCACAgC‐3′. To perform the western blotting assay, total protein was extracted using RIPA lysis buffer (Solarbio), and protein concentrations were measured using the BCA Protein Assay Kit (Beyotime). Following SDS‐PAGE, the protein was transferred onto PVDF membranes, and the membranes were blocked for 1 h at room temperature with 5% non‐fat dry milk. All antibodies were given as follows: CNIH4 Rabbit Antibody (1:1000, EPT‐A58610, Epigentek); Slug Rabbit Antibody (1:1000, 9585, CST); ZEB1 Rabbit Antibody (1:1000, 3396, CST); E‐Cadherin Rabbit Antibody (1:1000, 3195, CST); TWIST1 Rabbit Antibody (1:1000, AF4009, Affinity); BMI1 Rabbit Antibody (1:1000, 10,823, Proteintech); CD44 Rabbit Antibody (1:1000, 15,675, Proteintech); CD133 Rabbit Antibody (1:1000, 18,495, Proteintech); β‐actin Rabbit Antibody (1:3000, AF7018, Affinity); Goat anti‐rabbit IgG‐HRP (1:3000,7074, CST). All results were visualized using the ECL reagent kit (Tanon).

### 2.7 Cell Counting Kit‐8 (CCK‐8) assay

Cell proliferation of U87 and U251 cells was determined using the Cell Counting Kit‐8 (Dojindo). In three replicate wells of a 96‐well plate, 1 × 10^3^ cells were cultured in a medium containing 10% FBS in a volume of 100 μL per well. 10 μL of CCK‐8 reagent was then mixed with 90 μL of DMEM to create a working solution, and 100 μL of which was added to each well and incubated for 1.5 h before testing.^14^ which assay was performed at 24, 48, 72, and 96 h, respectively. As for TMZ sensitivity test, cells were transferred to a serum‐free medium while reaching a certain confluence and then treated with 50, 100, 200, and 300 μM TMZ for 24 h.

### 2.8 Cell migration and invasion assays

Cell migration was conducted using chambers made of polyethylene terephthalate (PET) with an 8 μm porosity (Corning). On uncoated filters in the upper chambers, the same number of tumor cells (1 × 10^4^ cells per well) were planted in serum‐free DMEM. In the bottom chambers, DMEM containing 10% FBS was introduced. Apical cells were removed with cotton swabs after 24 h of incubation, membranes were preserved in 100% methanol for 15 mins and then stained for 5 mins with Giemsa (Jiancheng). For invasion assays, 50 μL of diluted Matrigel solution (BD) was pre‐coated in the upper chambers as previously mentioned.^15^

### 2.9 Spheroid formation assay

Cell sphere formation capacity was determined using 6‐well ultra‐low attachment plates with 5 × 10^3^ cells per well cultured in serum‐free DMEM/F12 medium supplemented with B27 (1:50, Gibco), EGF (10 ng/mL, Proterintech) and bFGF (20 ng/mL, Proteintech). Fresh medium was introduced every 3 days. The total number of tumor spheres (diameter > 100 μm) was counted following 2 weeks of incubation.

### 2.10 Flow cytometry apoptosis assay

A total of 5 × 10^3^ cells were trypsinized without EDTA, washed twice with PBS, and stained with Annexin V‐FITC or PI Staining Solution (Vazyme) for 10 mins at room temperature as described in the user manual. The percentage of apoptotic cells was determined by flow cytometry.

### 2.11 Intracranial xenograft model and bioluminescent imaging (BLI)

1 × 10^5^ U87 cells were injected into the lateral ventricle of each 6–8 weeks‐old female Balb/c nude mouse as previously described.^16^ Luciferase images were captured using the In Vivo Imaging System (IVIS) at weeks 1 to 5 after injection. Each mouse was treated with 100 μL 15 mg/mL D‐Luciferin (Promega) intraperitoneally and examined after 10 mins.

### 2.12 Estimating tumor microenvironment (TME) infiltrations

Tumor Immune Estimation Resource (TIMER, https://cistrome.shinyapps.io/timer/) was systematically applied to estimate immune infiltration levels using TCGA expression profiles.^17^ Based on the expression of specific marker genes in different cancers, this tool uses deconvolution statistics to infer the quantity of six tumor‐infiltrating immune cells (TIICs), including CD8+ T cells, CD4+ T cells, B cells, macrophages, dendritic cells, and neutrophils. We examined the expression distribution of different immune cells in two clusters derived from vesicle trafficking‐related gene set. Additionally, correlations between CNIH4 expression and various infiltrating immune cell types were also explored.

### 2.13 Chemotherapy sensitivity and immunotherapy response prediction

Temozolomide half‐maximal inhibitory concentrations (IC50) were determined using Genomics of Drug Sensitivity in Cancer (GDSC, https://www.cancerrxgene.org/). The pRRophetic package was used to calculate the chemical sensitivity prediction of each glioma sample.^18^ Moreover, Tumor Immune Dysfunction and Exclusion (TIDE, http://tide.dfci.harvard.edu/), a reliable online algorithm suitable for predicting immunotherapeutic responses, was employed for the estimations.^19^

### 2.14 Statistical analysis

Statistical analysis and image generation were carried out using the R programming language (version 4.1.2) and the GraphPad Prism program (version 9.0). Wilcox t‐tests were utilized to compare differences between groups. Pearson's tests or Fisher's exact tests were used to explore the correlation between CNIH4 expression and clinicopathological variables. LASSO regression analysis, as well as univariate and multivariate Cox analyses, were employed for selecting independent variants. The Kaplan–Meier survival curve and the receiver operating characteristic (ROC) curve were used to evaluate the survival distributions. Statistical significance was defined as a two‐sided *p*‐value <0.05 (*p < 0.05, **p < 0.01, or ***p < 0.001).

## 3 RESULTS

### 3.1 Identification of vesicle trafficking‐related candidate in glioma

**FIGURE 1:**
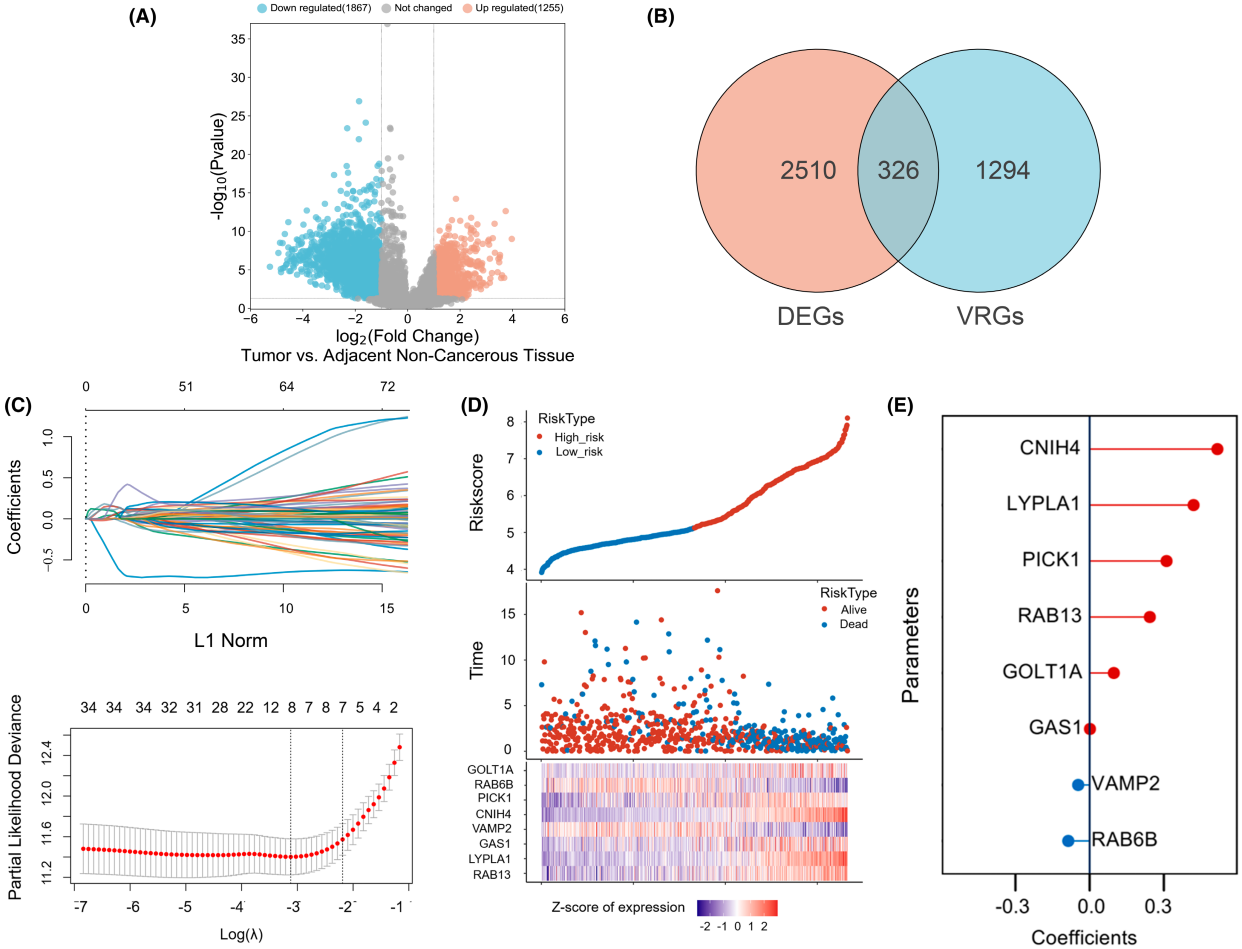
Identification of vesicle trafficking‐related candidates in glioma. (A) Volcano plot showing DEGs between glioma and adjacent non‐tumor tissues based on TCGA database. (B) Venn diagram determining the common genes between DEGs and VRGs. (C) LASSO coefficient profiles show the most relevant parameters determined by the optimal lambda in the upper panel and tuning parameter selection in the lower panel. (D) Prognostic curves and scatter plots show the correlation between risk score and survival status for each glioma patient. (E) Risk factors ranked by forest plot.

Using a cutoff of |log2fold change (FC)| > 2 and adj *p*‐value <0.05, we screened 2510 differentially expressed mRNAs (1867 down‐regulated and 1294 up‐regulated) in TCGA training cohort (666 glioma samples and 5 adjacent non‐tumor samples) and 326 of which were verified as VRGs according to the molecular signatures database (MSigDB) (Figure 1A,B). After eliminating the collinearity of genes, a VRG risk model was constructed using the LASSO‐Cox regression algorithm (Figure 1C). Each glioma patient was assigned a risk score according to the following formula: risk score = (0.2433)*RAB13 + (0.4201)*LYPLA1 + (0.0013)*GAS1 + (−0.0463)*VAMP2 + (0.5165)*CNIH4 + (0.3109)*PICK1 + (−0.0864)*RAB6B + (0.0978)*GOLT1.

X‐tile software was then used to generate the optimal cutoff for the risk score. Based on this cutoff value, TCGA patients were equally divided into low‐ and high‐risk groups. The prognostic curves and scatter plots suggested that the risk score and survival of each glioma patient were closely related, and most deaths were mainly distributed in the high‐risk group (Figure 1D). In addition, the forest plot showed that CNIH4 had the highest risk coefficient among all 8 VRGs, and was, therefore, selected as the target in the follow‐up study (Figure 1E).

### 3.2 CNIH4 is upregulated in glioma tissues, GSCs, and TMZ‐resistant cell lines

**FIGURE 2:**
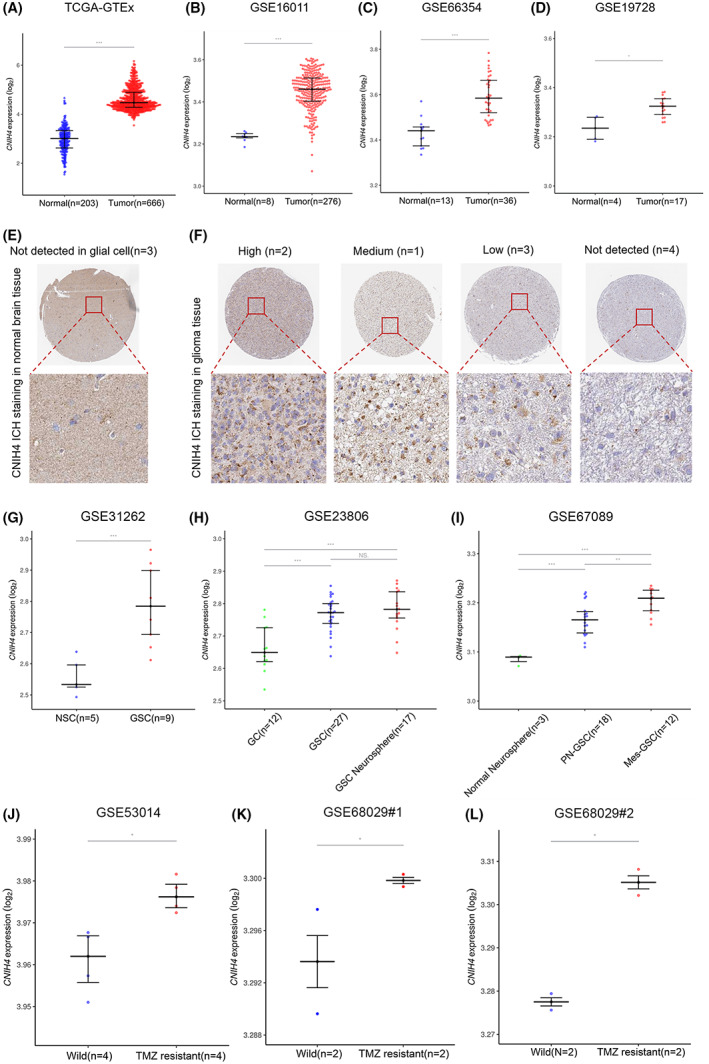
CNIH4 was aberrantly overexpressed in glioma tissues and cell lines. (A–D) CNIH4 mRNA expression pattern in normal brain and glioma tissues based on TCGA‐GTEx (Normal = 203, Tumor = 666), GSE16011 (Normal = 8, Tumor = 276), GSE66354 (Normal = 13, Tumor = 36), and GSE19728 (Normal = 4, Tumor = 17) datasets. Representative ICH images of CNIH4 in normal brain tissues (E) and glioma tissues (F). (G) Comparison of CNIH4 expression between Neural Stem Cell (NSC, *n* = 5), and Glioma Stem Cell (GSC, *n* = 9). (H) Comparison of CNIH4 expression among Glioma Cell (GC, *n* = 12), Glioma Stem Cell (GSC, *n* = 27), and Glioma Stem Cell Neurosphere (*n* = 17). (I) Comparison of CNIH4 expression among Normal Neurosphere, Proneural Glioma Stem Cell (PN‐GSC, *n* = 18), and Mesenchymal Glioma Stem Cell (Mes‐GSC, *n* = 12). (J–L) Comparison of CNIH4 expression between wild and TMZ‐resistant cell lines based on GEO datasets (GSE53014, GSE68029). *p < 0.05, **p < 0.01, ***p < 0.001, ns: no statistically significant.

CNIH4 mRNA expression was evaluated in normal brain and tumor tissues using the TGCA‐GTEx integrated dataset (*n* = 869). CNIH4 was revealed to be highly expressed in gliomas, and similar results were validated in GSE16011 (*n* = 284), GSE66354 (*n* = 49), and GSE19728 (*n* = 21) microarrays (Figure 2A–D). Further exploration of CNIH4 protein expression level was assessed using HPA staining data. While CNIH4 staining was not observed in glial cells in normal brain tissue (Figure 2E), a significant number of glioma tissues exhibited high (2/10), medium (1/10), or low (4/10) CNIH4 staining (Figure 2F). Surprisingly, we found high CNIH4 levels in GSC and GSC‐derived neurosphere compared to glioma cell (GC), neural stem cell (NSC), and NSC‐derived neurosphere from three independent validation cohorts (GSE31262, GSE23806, and GSE67089) (Figure 2G–I). In addition, GSC with mesenchymal (Mes) subtypes accumulated more CNIH4 than those with proneural subtypes (Figure 2I). A significant increase in CNIH4 mRNA was also observed in TMZ‐resistant glioma cells derived from two GEO microarray data cohorts (GSE53014 and GSE68029) (Figure 2J–L), implying its role in chemoresistance. The above findings collectively suggested CNIH4 as a novel GSCs‐related molecule that was considerably elevated at both the tissue and cellular levels.

### 3.3 Correlation between CNIH4 expression with Clinicopathologic Phenotypes of Glioma

**FIGURE 3:**
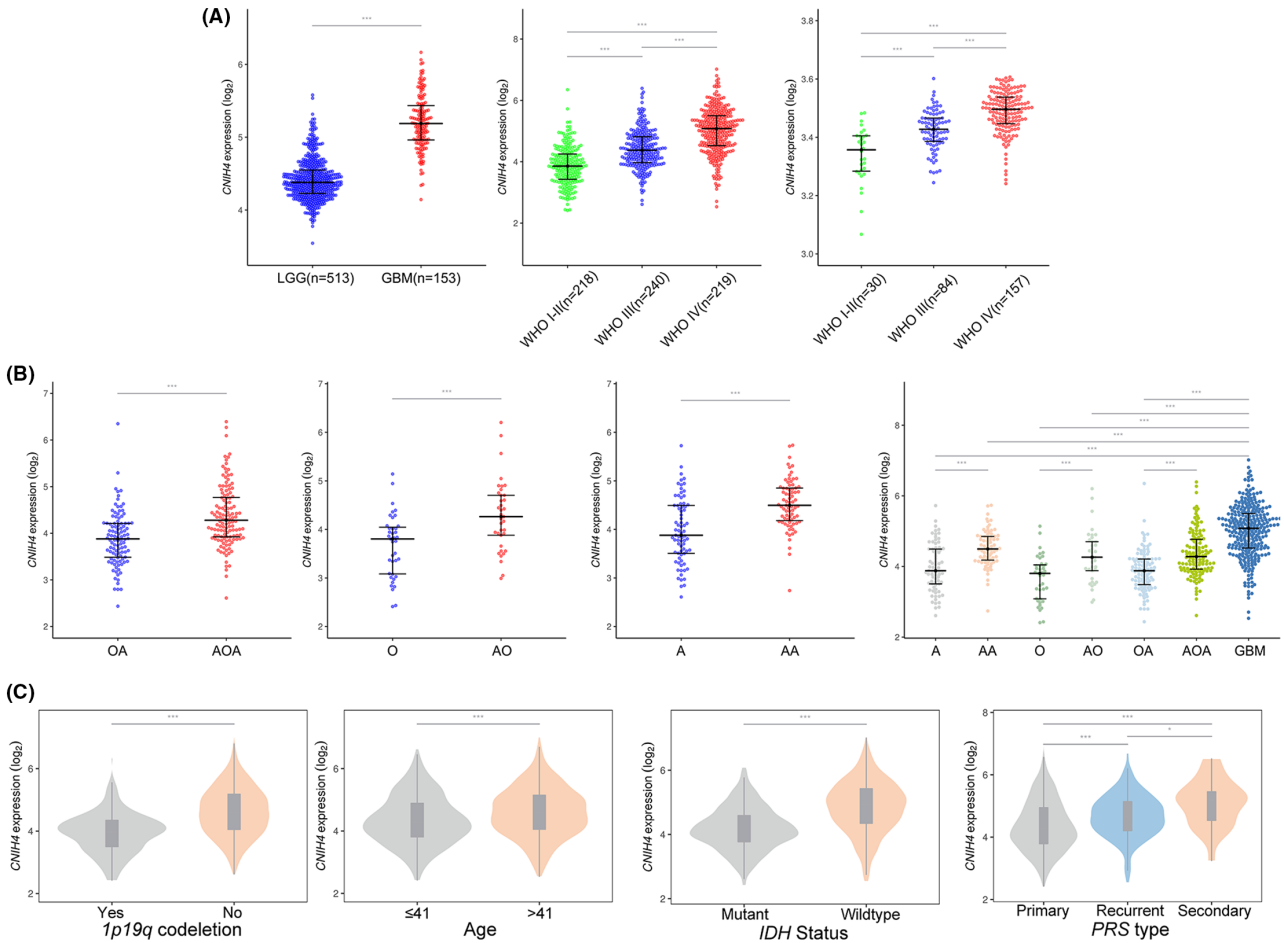
Clinicopathological relevance of CNIH4 in glioma. (A) CNIH4 mRNA expression pattern in different WHO grades of gliomas in TCGA, CGGA sequencing, and GEO microarray datasets. (B) Comparison of CNIH4 expression pattern among pathological isoforms (O, A, OA) and their malignant transformed subtypes (AO, AA, AOA, GBM). (C) CNIH4 expression patterns and their correlation with clinicopathological parameters including age, 1p19q codeletion, IDH status, and PRS type in CGGA database. A, Astrocytomas; AA, Anaplastic astrocytomas; AO, Anaplastic oligodendrogliomas; AOA, Anaplastic oligoastrocytomas; GBM, Glioblastomas; O, Oligodendrogliomas; OA, Oligoastrocytomas; PRS, Primary, Recurrent, or Secondary recurrent tumor. *p < 0.05, **p < 0.01, ***p < 0.001, ns: no statistically significant.

We examined CNIH4 expression levels across different grades in TCGA (including LGG and GBM, *n* = 666). Upregulated CNIH4 expression was more prevalent in advanced gliomas (Figure 3A). This result was validated using CGGA sequencing (WHO grade 2–4, *n* = 749) and GEO microarray (WHO grade 1–4, *n* = 271) datasets. Furthermore, we assessed CNIH4 expression across different histological subtypes and found CNIH4 was significantly higher in anaplastic glioma subtypes such as anaplastic astrocytomas, anaplastic oligodendrogliomas, and anaplastic oligoastrocytomas, all of which were classified as malignant forms of glioma (Figure 3B). Notably, CNIH4 had the prominent expression level in the GBM subtype. Clinicopathological parameters based on CNIH4 expression groups were further assessed in the CGGA cohort (*n* = 749). Age, PRS type, IDH mutation, and 1p/19q codeletion status all differed significantly (Figure 3C, Table 1). These characteristics have previously been identified as adverse clinical variables associated with poor OS, implying that CNIH4 significantly influences glioma progression.

**TABLE 1:** Correlation of CNIH4 expression with clinical parameters in patients with glioma based on CGGA dataset.

	Total (*N* = 749)	CNIH4 Expression		*p*‐value
		High (*N* = 446)	Low (*N* = 303)	
Age (year)				
≤41	342 (45.7%)	178 (39.9%)	164 (54.1%)	<0.001
>41	407 (54.3%)	268 (60.1%)	139 (45.9%)	
Gender				
Male	442 (59.0%)	266 (59.6%)	176 (58.1%)	0.727
Female	307 (41.0%)	180 (40.4%)	127 (41.9%)	
PRS type				
Primary	502 (67.0%)	261 (58.5%)	241 (79.5%)	<0.001
Recurrent	222 (29.6%)	163 (36.5%)	59 (19.5%)	
Secondary	25 (3.3%)	22 (4.9%)	3 (1.0%)	
WHO Grade				
II	218 (29.1%)	56 (12.6%)	162 (53.5%)	<0.001
III	240 (32.0%)	140 (31.4%)	100 (33.0%)	
IV	291 (38.9%)	250 (56.1%)	41 (13.5%)	
Radiotherapy				
Yes	625 (83.4%)	373 (83.6%)	252 (83.2%)	0.946
No	124 (16.6%)	73 (16.4%)	51 (16.8%)	
Chemotherapy				
Yes	520 (69.4%)	344 (77.1%)	176 (58.1%)	<0.001
No	229 (30.6%)	102 (22.9%)	127 (41.9%)	
IDH Status				
Wildtype	339 (45.3%)	265 (59.4%)	74 (24.4%)	<0.001
Mutant	410 (54.7%)	181 (40.6%)	229 (75.6%)	
1p19q codeletion				
Yes	155 (20.7%)	50 (11.2%)	105 (34.7%)	<0.001
No	594 (79.3%)	396 (88.8%)	198 (65.3%)	

### 3.4 High CNIH4 levels predict unfavorable outcome and serve as a superior indicator

**FIGURE 4:**
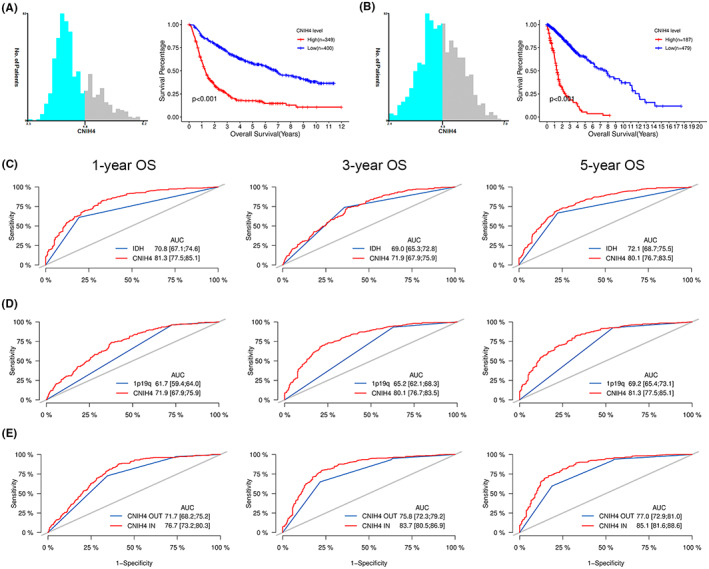
Survival outcome analysis of glioma patients in CGGA validation cohort. Kaplan–Meier (K‐M) survival curves showing the overall survival (OS) based on X‐tile plots cut‐off point in CGGA (A) and TCGA (B) sequencing databases. (C) Receiver operating characteristic (ROC) curves showing the significance of CNIH4 in predicting 1‐, 3‐, 5‐ year survival compared with IDH status (D) and 1p/19q codeletion. (E) ROC curves showing the predictive significance of prognostic factors combination of “IDH status and 1p/19q codeletion” compared with prognostic factors combination of “CNIH4 level, IDH status, and 1p/19q codeletion”.

A Kaplan–Meier (K‐M) analysis was performed on 749 and 869 patients from the CGGA and TCGA sequencing datasets, respectively, to explore the relationship between CNIH4 expression and survival outcomes. The optimal OS thresholds were assessed using X‐tile software (version 1.0.0) before analysis.^20^ The survival time of patients with higher CNIH4 expression was significantly shorter in both two cohorts (Figure 4A,B). Given the present limitations of glioma candidates, we further explored the predictive role of CNIH4 in comparison with established indicators (IDH mutation and 1p/19q codeletion). As shown in Figure 4C,D, the AUC areas for CNIH4 level in predicting 1 to 5 years of survival in the CGGA dataset were 71.9, 80.1, and 81.3, which were substantially greater than “IDH status” (69.0, 72.1, and 70.8) and “1p/19q codeletion status” (61.7, 65.2 and 69.2). A subsequent analysis comparing the AUC values of “include CNIH4 level” and “exclude CNIH4 level” found that combinations of indicators integrating CNIH4, IDH, and 1p/19q have superior predictive performance than that integrating IDH and 19/19q (76.7, 83.7 and 85.1 vs. 71.7, 75.8 and 77.0) (Figure 4E). These results demonstrated that CNIH4 is a reliable indicator for predicting the OS of glioma and may serve as a promising candidate.

### 3.5 Validation of CNIH4 as an independent risk factor for glioma patients

**FIGURE 5:**
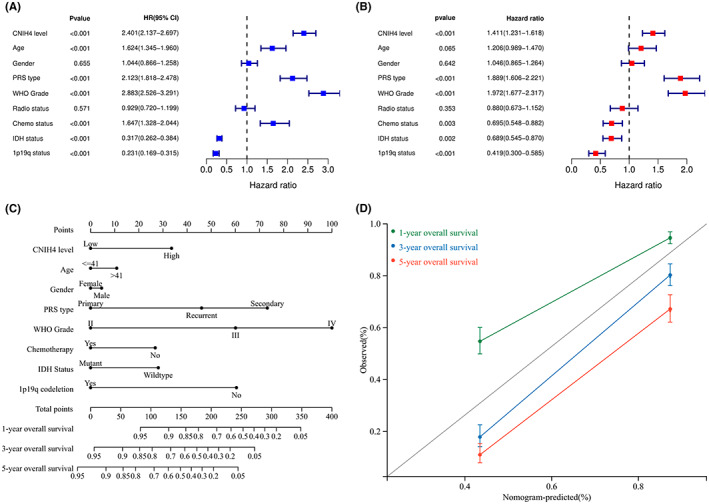
Validation of the independent prognostic significance of CNIH4 in glioma using the CGGA validation cohort. (A, B) Forest plots showing the results of Univariate Cox analysis (left) and multivariate Cox analysis (right) regarding OS of CNIH4 with other prognostic factors. (C) Prognostic nomogram predicting the 1‐, 3‐, and 5‐year survival risk of glioma patients. (D) The calibration curves of the nomogram for predicting 1‐, 3‐, and 5‐year overall survival.

To obtain a more precise assessment of CNIH4's prognostic value in glioma, both univariable (Figure 5A) and multivariable (Figure 5B) Cox regression was further utilized to explore independence regarding OS in glioma patients from the CGGA validation cohort. CNIH4 overexpression contributed independently to poor OS following the application of univariable (Odds ratio 2.401 with 95% CI [2.137–2.697], *p* < 0.001) and multivariable (Odds ratio 1.411 with 95% [CI 1.23–1.616], *p* < 0.001) analytic models. The following nomogram based on the results above showed a great contribution of CNIH4 to the probability of survival (Figure 5C). As shown in Figure 5D, the nomogram‐predicted survival probability (1‐, 3‐, and 5‐year OS) closely matched the actual results.

### 3.6 Functional enrichment analysis revealed the stemness‐related processes associated with CNIH4 in glioma

**FIGURE 6:**
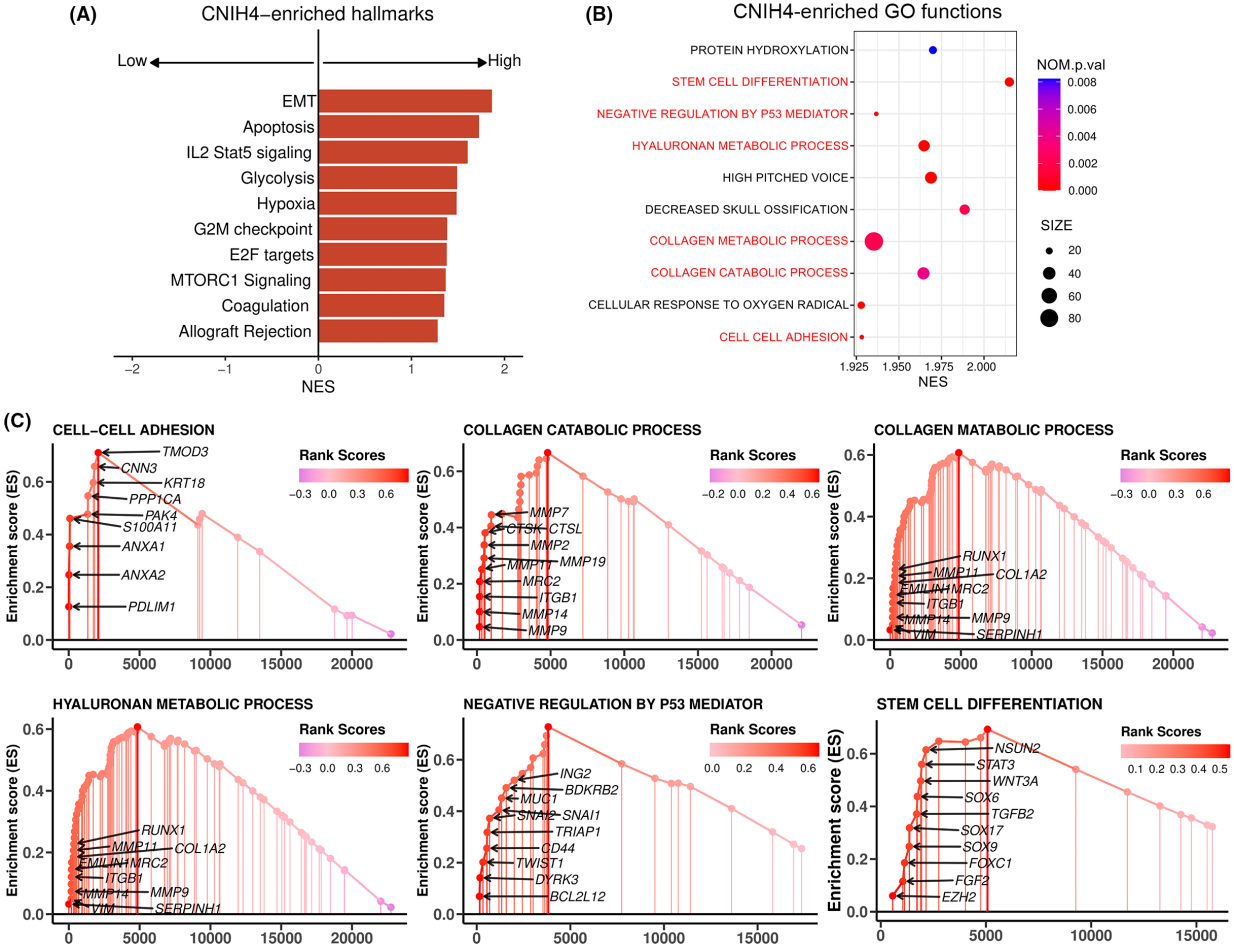
Biological functions related to the expression of CNIH4. (A) The top 10 tumor‐related hallmarks and pathways that were significantly enriched in patients with high level of CNIH4 expression as determined through GSEA analysis in the CGGA sequencing database. (B) The top 10 signaling pathways ranked by NES in the GO analysis. (C) Related GSEA plots with annotated hub genes based on GO analysis.

To elucidate the functional consequences of abnormally expressed CNIH4 in glioma, GSEA was performed in the CGGA sequencing dataset. Notably, the relative genes in the up‐CNIH4 subtype were significantly enriched in characteristic processes such as EMT and apoptosis (Figure 6A). Tumor stroma signaling pathways, stem cell differentiation, as well as anti‐apoptotic pathways were positively enriched through GO analysis and were summarized as follows: cell–cell adhesion (NES = 1.92, NOM *p*‐value <0.001), collagen catabolic process (NES = 1.96, NOM *p*‐value = 0.004), collagen metabolic process (NES = 1.93, NOM *p*‐value = 0.002), hyaluronan metabolic process (NES = 1.96, NOM *p*‐value <0.001), negative regulation by p53 mediator (NES = 2.01, NOM *p*‐value <0.001), and stem cell differentiation (NES = 1.93, NOM *p*‐value <0.001) (Figure 6B,C). In addition, hallmarks related to cell cycle proliferation such as G2M checkpoints, E2F targets, and MTORC1 signaling are substantially enriched in the up‐CNIH4 subtype, demonstrating that CNIH4 influenced cell division. Moreover, the potential correlations of CNIH4 with CSC markers were further detected and were declared to be strong (Figure S1). All findings suggest that CNIH4 is closely related to stem‐like characteristics of glioma.

### 3.7 Downregulation of CNIH4 expression suppressed the stem cell‐like characteristics of glioma in vitro and in vivo

**FIGURE 7:**
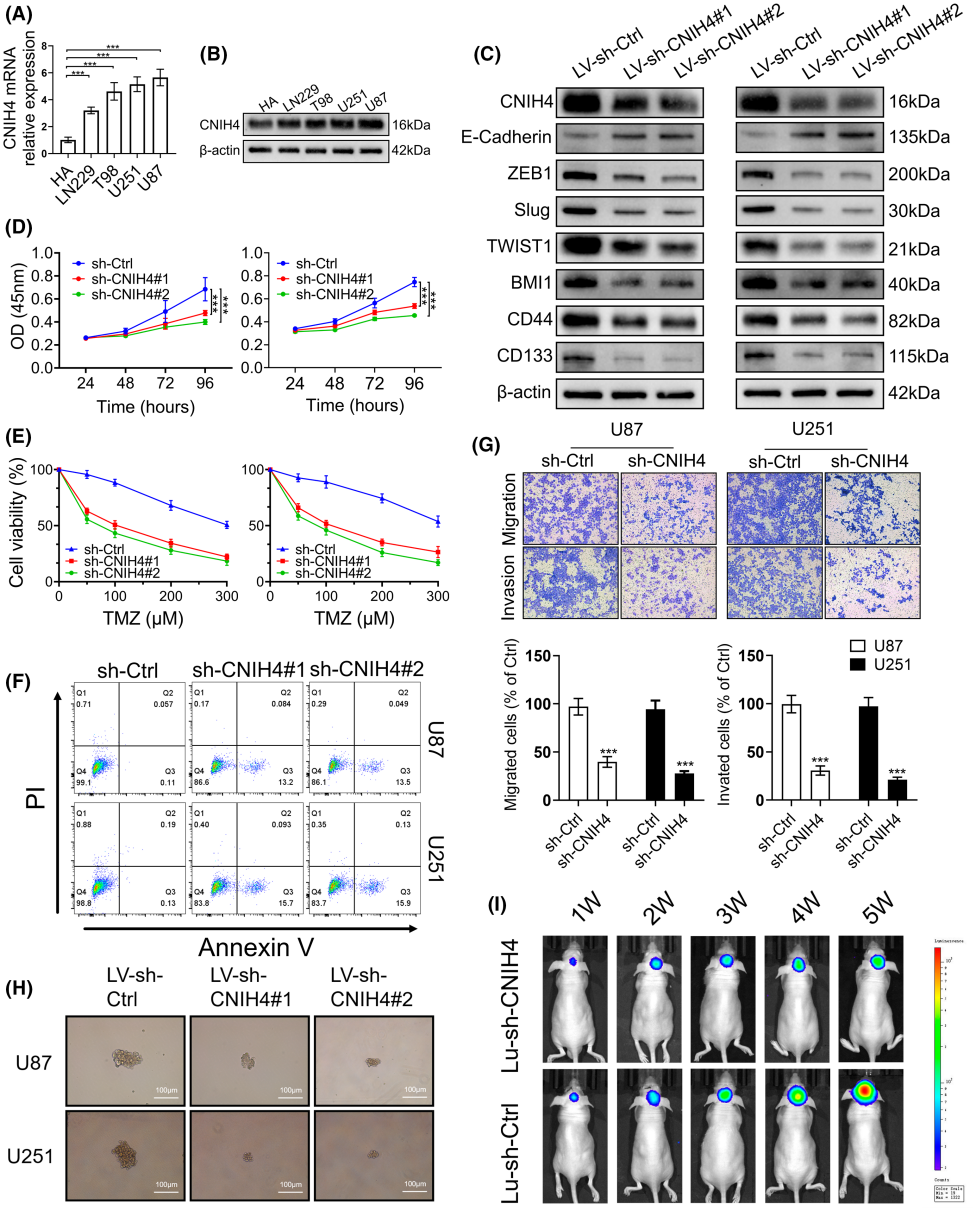
Stem cell‐like characteristics of CNIH4 expression in glioma cells. The basal CNIH4 expression was measured through real‐time quantitative PCR (RT‐PCR) technique (A) and western blot analysis (B) in human normal astrocytes (HA) and glioma cell lines. (C) Western blot results showing expression of EMT‐related protein and stem cell markers in U87 and U251 cells after the transfection of CNIH4 shRNAs (#1 and #2) or a sh‐Ctrl as a contrl. (D) Cell proliferation following CNIH4 knockdown in U87 and U251 cells as determined by CCK‐8 assays; (E) TMZ toxicity following CNIH4 knockdown in U87 and U251 cells as determined by CCK‐8 assays. (F) Representative images showing apoptosis of U87 and U251 cells after CNIH4 silencing as determined by Annexin V/PI staining and flow cytometry analyses. (G) Cell migration and invasion following CNIH4 knockdown in U87 and U251 cells as detected by transwell assays. (H) Representative images of U87 and U251 neurospheres transduced with sh‐CNIH4 or a negative control (sh‐Ctrl). (I) Representative images showing the proliferation of U87 cells after CNIH4 silencing as determined by in vivo imaging system (IVIS) performed 1 to 5 weeks post‐cell injection. *p < 0.05, **p < 0.01, ***p < 0.001, ns: no statistically significant.

The expression level of CNIH4 expression was measured in human astrocytes (HA) and human glioma (LN229, T98, U251, and U87) cell lines. RT‐PCR and western blot analyses showed a relatively increased level of CNIH4 in U87 and U251, but lower expression in HA (Figure 7A,B). Subsequently, LV‐sh‐CNIH4 (ctrl, #1, #2) was transfected into U87 and U251 cells to downregulate CNIH4 expression. Compared to the control cells, the protein level of EMT transcriptional markers ZEB1, Slug, TWIST1 and stem cell markers BMI1, CD44, CD133 was significantly decreased in CNIH4 knockdown cells, whereas the expression of epithelial marker E‐cadherin was up‐regulated, indicating that CNIH4 affected stemness‐related genes (Figure 7C). CCK‐8 assays showed that CNIH4 silencing dramatically inhibited cell proliferation compared to control cells while enhancing sensitivity treated with TMZ chemotherapy (Figure 7D,E). Moreover, Flow cytometric analysis indicated that CNIH4 knockdown increased the rate of apoptotic cell death (Figure 7F). In transwell assays, knockdown of CNIH4 impaired glioma cell migration and invasion (Figure 7G). In addition, it inhibited the sphere‐forming ability of cells, suggesting the impairment of self‐renewal capacity (Figure 7H). In a further experiment in vivo, we found that U87 cells labeled with luciferase stably expressing shCNIH4 (U87‐Lu‐shCNIH4) grew much slower in nude mice than in the control group (U87‐Lu‐shCtrl) as determined by fluorescence tests, indicating the impairment of tumorigenesis (Figure 1). Overall, all findings demonstrated that CNIH4 influenced stem‐cell‐related properties in glioma cells.

### 3.8 Mutational landscape, chemotherapy sensitivity and immunotherapy response of CNIH4 in glioma

**FIGURE 8:**
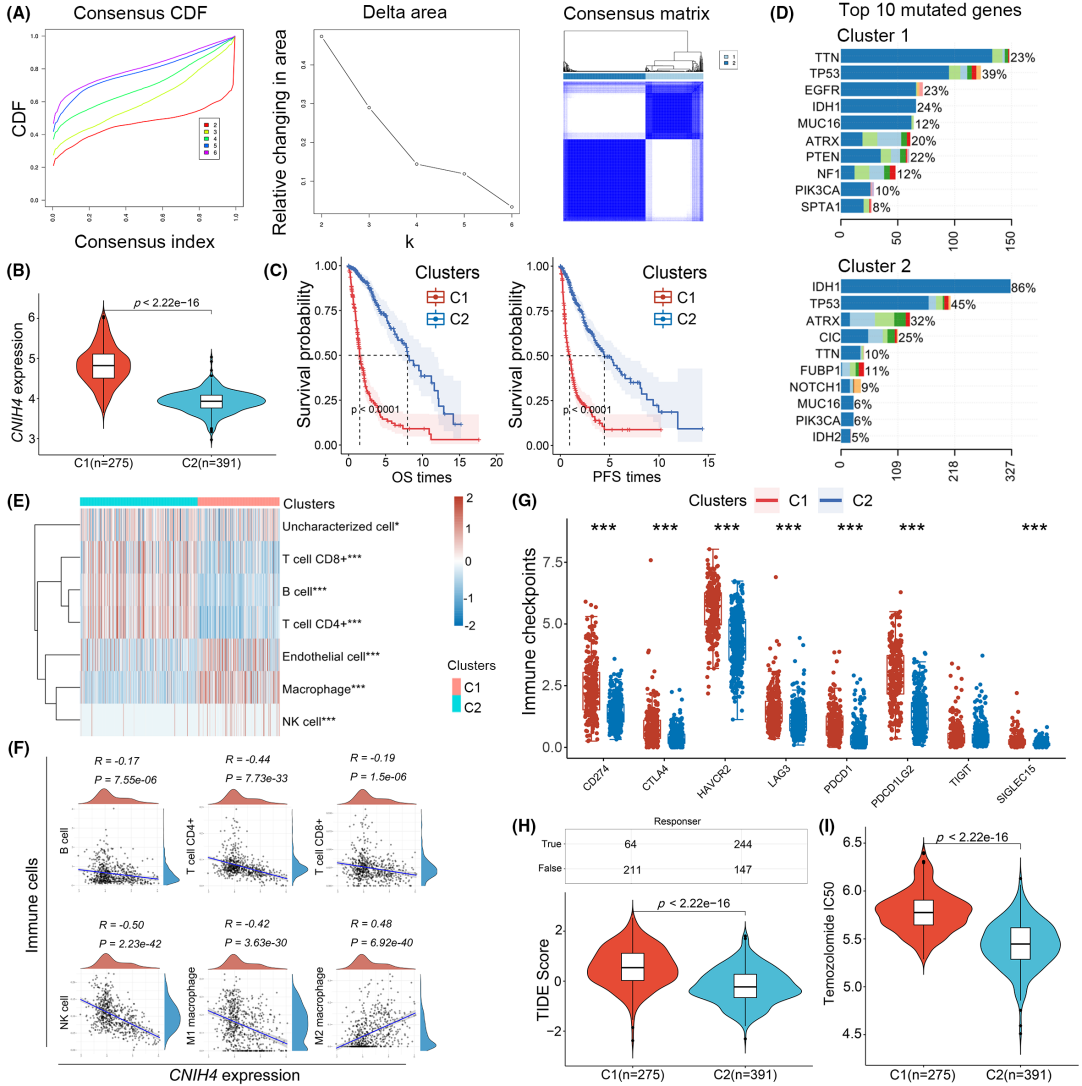
Mutation landscape, immunotherapy response, and chemotherapy sensitivity of CNIH4‐enriched subtypes in glioma. (A) Consensus clustering identified two distinct clusters based on vesicle trafficking‐related gene set. (B) CNIH4 expression pattern in the two clusters. (C) Kaplan–Meier curves showing the OS and PFS of glioma patients. (D) Histogram showing the top 10 mutational sites. (E) Heatmap showing differences in 6 immune cells between the two clusters. (F) Correlation of CNIH4 expression pattern with 6 immune cells. (G) Box plot showing differences in 8 immune checkpoints between the two clusters. (H) Distribution of responders and non‐responders to immunotherapy between the two clusters estimated by the TIDE algorithm. (I) Box plot for the estimated IC50 of TMZ chemotherapy between clusters.

Based on a vesicle trafficking‐related gene set containing 1620 VRGs, unsupervised clustering was performed with the ConsensusClusterPlus package to classify glioma patients into two distinct clusters (C1‐2, Figure 8A). It was observed that C1 typically accumulated up‐regulated CNIH4 expression (Figure 8B). Kaplan–Meier analysis revealed that C1 is indicative of more undesirable OS and PFS outcomes compared with C2 (Figure 8C). We also evaluated the prevalence of genomic mutations in two subpopulations and found most regions in C1 had a higher incidence of mutations, while the mutation rate in C2 was generally lower except IDH1, suggesting a completely different mutational status (Figure 8D). TIMER analysis was then performed to elucidate the TIME landscape across clusters. As shown in Figure 8E,F, C1 displayed an immunosuppressive subtype characterized by high macrophage infiltration, while C2 exhibited higher levels of anti‐tumor TIME components like CD8+ T cells, CD4+ T cells, and B cells. As a result of the analysis of CNIH4 expression and immune cells, it was found that CNIH4 was positively correlated with immunosuppressive cells (M2 macrophages, *R* = 0.48), but negatively correlated with anti‐tumor cells (CD4+ T cells, CD8+ T cells, B cells, NK cells, and M1 macrophages; *R* = ‐0.44, *R* = −0.19, *R* = −0.17, *R* = −0.50, *R* = −0.42, respectively), suggesting its role in the antitumor immunity. Furthermore, C1 showed relatively high expression of immune checkpoints and higher TIDE scores compared with C2 (Figure 8G,H). As a result, 244 of 391 patients (62.4%) in cluster 2 were predicted to benefit from immunotherapy while those in cluster 1 were estimated to derive limited benefit (61 of 276, 22.1%). Surgery combined with TMZ therapy has been established as the standard treatment for glioma patients. Thus, we estimated IC50 values indicative of chemosensitivity between groups. In Figure 8I, we noted that the IC50 value of TMZ was significantly higher in C1 compared with C2, implying that the CNIH4‐enriched subtype may display less sensitivity to current chemotherapy.

## 4 DISCUSSION

Gliomas are the most infiltrative and deadliest tumor type in the central nervous system.^1^ Despite the application of innovative strategies, the long‐term survival rate of most advanced gliomas remains unsatisfactory.^2^, ^21^ Molecular diagnostic techniques have been remarkably successful and variety of tumor‐specific genetic alterations in gliomas were routinely applied in diagnosis. For instance, isocitrate dehydrogenase (IDH) mutations and chromosome 1p/19q codeletion as positive prognostic markers in adult patients, indicating longer survival^22^; H3F3A alterations predict worse outcomes in pediatric glioma patients^23^; TERT promoter mutations and EGFR alterations lead to IDH‐wildtype astrocytoma reaching the highest tumor grade with the worst prognosis.^24^ However, many patients still end up dying from tumor recurrence for standard regimens lacking sufficient specificity. Thus, more specific targets are urgently needed in this field.

Growing evidence reveal that vesicle trafficking, including the release of extracellular microvesicles, is a highly important process involved in many aspects of human diseases, such as neurotransmitter release, hormone secretion, and natural immune regulation.^25^ In high‐grade gliomas, vesicles participate in a variety of processes, including tumor growth, migration/invasion, angiogenesis, and even immunological functions.^26^ Recent studies verified that vesicle‐trafficking‐related AMPA receptors are actively involved in signaling and communications related to neuron‐glioma synapses, and thus promote glioma xenograft growth.^27^, ^28^ These suggested that VRGs are closely relevant to glioma progression and have the potential to serve as survival targets. CNIH4, known as Cornichon family AMPA receptor auxiliary protein 4, is essential for transporting vesicles from their synthetic site to the function site, previously reported to be amplified in multiple human malignancies and predicted poor prognosis.^6^, ^7^, ^8^ A relevant study confirmed that CNIH4 increased the metastatic activity in patients with colon cancer by forming a positive feedback loop involving TMED9, GLI1, and TGFα.^7^ Furthermore, knockdown of CNIH4 suppressed the proliferation of gastric cancer cells.^9^ However, the clinical applications and functional studies of CNIH4 in glioma are still limited.

In this investigation, we screened Cornichon family AMPA receptor auxiliary protein 4 (CNIH4) from VRGs following LASSO‐Cox regression algorithms and then assessed the basal expression at both the mRNA and protein levels from multi‐scaled database analysis. We noted that CNIH4 was up‐regulated in glioma tissues, GSCs, and further elevated in TMZ‐resistant cell lines. It is commonly associated with tumor grades and considerably increased in high‐grade glioma. Moreover, up‐regulated CNIH4 was positively correlated with undesirable clinicopathological characteristics. Univariate and multivariate Cox regression analyses revealed that CNIH4 independently predicted the prognosis of patients. Notably, ROC curves suggested CNIH4 was a superior indicator to typical measures (IDH mutation and 1p/19q codeletion), demonstrating the significant predictive ability for survival. These results above revealed that CNIH4 expression is tightly associated with the malignant progression of glioma.

We also utilized GSEA to determine the functional relevance of CNIH4 expression in glioma. The results suggested a significant correlation of CNIH4 with stem cell‐like processes such as EMT and apoptosis. GO analysis revealed stem cell differentiation and stroma metabolic processes including cell–cell adhesion, collagen catabolic process, collagen metabolic process, and hyaluronan metabolic process, as well as p53‐mediated apoptotic pathway was significantly enriched. Recent research indicated the emergence of CSCs occurs partially as a result of EMT through crosstalk with tumor stromal components.^29^, ^30^ Mechanistically, EMT can lead to the loss of cell polarity and intercellular adhesion, giving rise to stem cell invasive and migratory properties.^31^ It also stimulates anti‐apoptotic signaling, making CSCs resistant to chemotherapy.^32^ Moreover, as critical members of tumor stroma, hyaluronan, and collagen can preserve the normal phenotype of human endothelial cells and CSCs may induce the degradation of these components, thereby facilitating EMT response.^33^, ^34^ Relative molecules underlying these processes were explored as follows: matrix metalloproteinases (MMP1/2/7/9/11/14/19), transcription factors (SNAI1, SNAI2, and TWIST), CD44, ANXA1, ANXA2, and other inflammatory factors (NFBK1, TGFB1, and IL‐6), all of which were found to be positively correlated with CNIH4 expression. As a family of zinc endopeptidases, MMPs have demonstrated significant potential in degrading the extracellular matrix (ECM), consequently initiating EMT in cancer cells and promoting cell invasiveness.^35^ Additionally, the inflammatory environment may support tumors by stabilizing EMT via the NF‐kappaB‐induced/TGF‐beta mechanism.^36^, ^37^ Annexin A1 (ANXA1), an emerging candidate, has been used as an EMT regulator in several tumor types.^38^, ^39^ Moreover, CD44 was found to be expressed in malignant mesenchymal GSC (Mes‐GSC) and served as a Mes‐GSC marker during glioblastoma metastasis.^40^ Consistently, our study observed a similar result that CNIH4 was typically accumulated in the Mes‐GSC subtype. Therefore, we cautiously approach CNIH4 as a biomarker for the mesenchymal subtype of glioma stem cells.

Cancer stem cells (CSCs), a key feature of human cancers, play a critical role in triggering incomplete resections and tumor recurrences.^41^ This subpopulation has been directly related to aberrant proliferation, rapid migration, and aggressive invasion in most of the human cancers.^42^ To date, several CSC surface markers, including CD133, CD24, and CD44, have been reported in glioma.^43^ A recent founding revealed that BMI1 regulates CD133+ brain tumor‐initiating cells in human glioblastoma stem cells, emphasizing the importance of the CD133‐BMI1 circuit in stemness maintenance.^44^ We demonstrated that CNIH4 has a significant effect on GSC markers (CD144, CD44, and BMI1) via knocking down CNIH4 by using specific shRNAs, implying that CNIH4 as a definite therapeutic target in glioma stem cells. Moreover, CSCs have been implicated in the EMT process, in which non‐motile epithelial cells are transformed into a mesenchymal phenotype and hence gain invasive capabilities.^45^ Our present study confirmed that inhibiting CNIH4 decreased the expression of mesenchymal markers (SLUG, ZEB1, TWIST1) involved in EMT while increasing the expression of epithelial markers (E‐cadherin). These findings strongly support the key role of CNIH4 in glioma invasion. Also, the nature of GSC self‐renewal and differentiation capacity has been reported to contribute to drug resistance and tumorigenesis, which is regarded to be the primary reason for the failure of current malignant glioma therapies.^38^ In our investigation, we proved that CNIH4 influences self‐renewal potential in malignant glioma cells using a self‐formation assay. Besides, the regulatory effect towards aberrant proliferation was further detected via proliferation assay in vitro and xenograft models in vivo. In light of these findings, CNIH4 can be used as a stem cell‐like target for glioma.

Research evidence suggested that CSCs have immune‐modulatory capabilities, from which some immune properties might not be affected by host anticancer immunity.^46^ Besides, this subpopulation can interact with immune checkpoints and produce immune system suppressors to protect cancer cells from immune clearance, thereby facilitating tumor evasion.^47^ As a newly identified GSC‐related biomarker of CNIH4, its correlation with TIME required further exploration. In the current investigation, it is worth noting that CNIH4 correlates positively with immunosuppressive cells (M2 macrophages) and negatively with anti‐tumor immune cells (CD8+ T cells, CD4+ T cells, B cells, NK cells, and M1 macrophages), as well as an abundance of immune checkpoint molecules like CD274, HAVCR2, CTLA4, LAG3, SIGLECT15, and PDCD1. TIDE and pRRophetic algorithms revealed the CNIH4‐enriched subtype was associated with a lower immunotherapy response and sensitivity to TMZ treatment. To summarize, CNIH4 potentially plays roles in glioma TIME and may guide current glioma therapy.

This study extensively investigated the clinical application of CNIH4 in glioma, but the precise molecular mechanism that underlies its stemness via vesicle trafficking processes is unclear and warrants in‐depth study. More comprehensive in vitro experimental verification and in vivo models are imperative to support our findings. What is more, the expression profile of CNIH4 in real cultured GCSs may refine our research and provide more accurate diagnosis/prognosis predictions for patients.

## 5 CONCLUSION

The 8‐VRGs risk model and survival distribution analysis confirmed the independent prognostic role of CNIH4 in glioma. Bioinformatic analysis revealed that CNIH4 participates in glioma malignancy, stemness‐like processes, immunotherapy response, and chemotherapy sensitivity. Experimental approaches demonstrated that CNIH4 suppression dramatically enhanced TMZ sensitivity and apoptosis while inhibiting proliferation, migration/invasion, sphere‐formation, as well as tumorigenicity in glioma cells. Therefore, CNIH4 holds great promise as an independent predictive marker and a target for stem cell‐like therapy.

## AUTHOR CONTRIBUTIONS

**Zhen Fang:** Writing – original draft (equal); writing – review and editing (equal). **Fangen Kong:** Writing – original draft (equal); writing – review and editing (equal). **Jia Zeng:** Data curation (equal); formal analysis (equal). **Zichen Zhang:** Investigation (equal); methodology (equal); project administration (equal). **Yunzhi Wang:** Investigation (equal); methodology (equal); project administration (equal). **Yiping Wang:** Resources (equal); software (equal). **Jiajia Duan:** Resources (equal); software (equal). **Lei Chen:** Resources (equal); software (equal). **Jikai Wang:** Validation (equal); visualization (equal). **Fei Liu:** Conceptualization (equal); funding acquisition (equal).

## FUNDING INFORMATION

The National Natural Science Foundation of China (#81870944 to F.L) and the Science Technology Planning Project of Zhuhai, China (Grant No. 20191210E030073 to F.K) provided funding for this study.

## CONFLICT OF INTEREST STATEMENT

The authors declare no conflict of interest in this work.

## ETHICS STATEMENT

All animal experiments (BALB/c nude mice) were approved by the Scientific Ethics Committee of the Fifth Affiliated Hospital, Sun Yat‐sen University, Zhuhai, Guangdong, China.

## Supporting information


Figure S1

## Data Availability

The dataset profile contributed to this study can be downloaded here: http://www.cgga.org.cn/, https://www.ncbi.nlm.nih.gov/geo/, https://portal.gdc.cancer.gov/
