# Supplementary figures and images for: Integrated analysis based on vesicle trafficking‐related genes identifying CNIH4 as a novel therapeutic target for glioma
Source: Cancer Med. 2023 Apr 16;12(11):12943–59. doi: 10.1002/cam4.5947 (PMC10278472; doi:10.1002/cam4.5947)

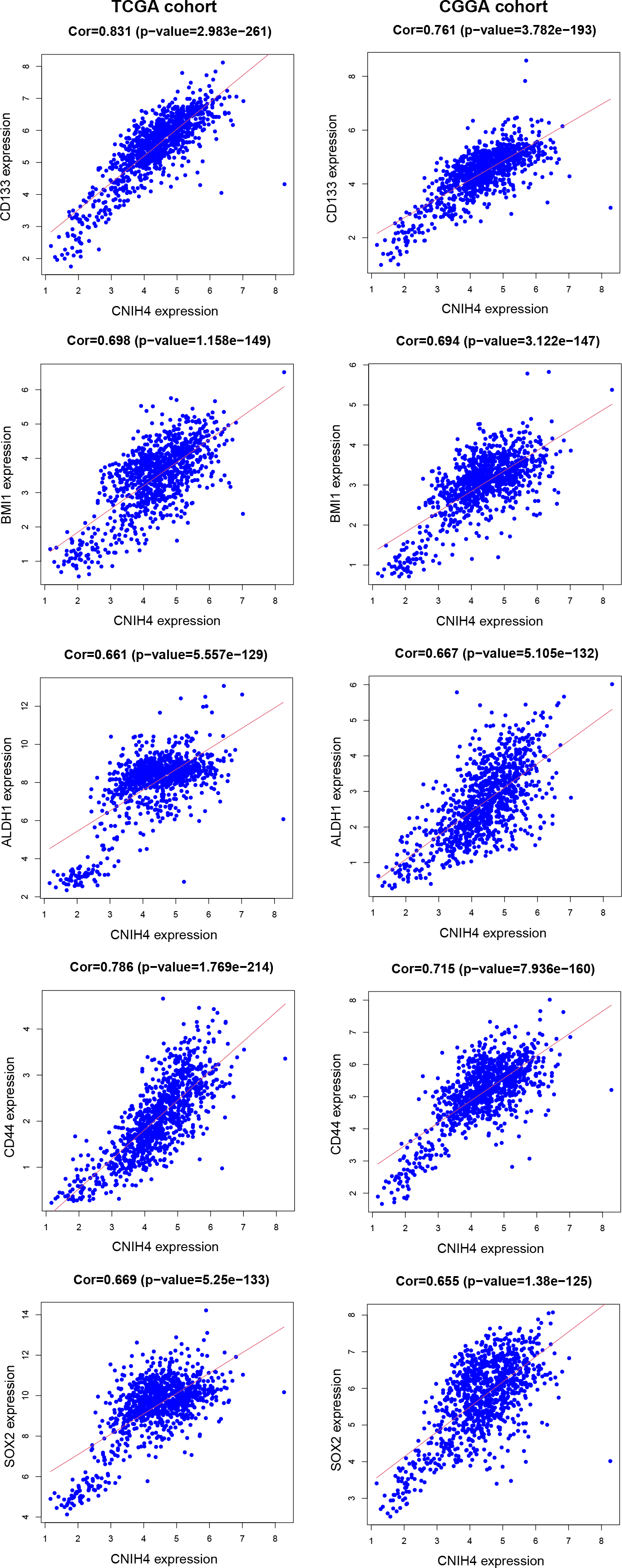

Supplement: Supplementary file 1 — Figure S1 [file CAM4-12-12943-s001.tif]
